# Emission Trade-Off between Isoprene and Other BVOC Components in *Pinus massoniana* Saplings May Be Regulated by Content of Chlorophylls, Starch and NSCs under Drought Stress

**DOI:** 10.3390/ijms24108946

**Published:** 2023-05-18

**Authors:** Runxia Huang, Tianning Zhang, Xiaogai Ge, Yonghui Cao, Zhengcai Li, Benzhi Zhou

**Affiliations:** 1 Research Institute of Subtropical Forestry, Chinese Academy of Forestry, Hangzhou 311400, China; 2 Qianjiangyuan Forest Ecosystem Research Station, National Forestry and Grassland Administration of China, Hangzhou 311400, China

**Keywords:** drought stress, biogenic volatile organic compounds, *Pinus massoniana* saplings, physiological characteristics

## Abstract

The aim of this work was to study the changes in the BVOCs emission rates and physiological mechanistic response of *Pinus massoniana* saplings in response to drought stress. Drought stress significantly reduced the emission rates of total BVOCs, monoterpenes, and sesquiterpenes, but had no significant effect on the emission rate of isoprene, which slightly increased under drought stress. A significant negative relationship was observed between the emission rates of total BVOCs, monoterpenes, and sesquiterpenes and the content of chlorophylls, starch, and NSCs, and a positive relationship was observed between the isoprene emission rate and the content of chlorophylls, starch, and NSCs, indicating different control mechanism over the emission of the different components of BVOCs. Under drought stress, the emission trade-off between isoprene and other BVOCs components may be driven by the content of chlorophylls, starch, and NSCs. Considering the inconsistent responses of the different components of BVOCs to drought stress for different plant species, close attention should be paid to the effect of drought stress and global change on plant BVOCs emissions in the future.

## 1. Introduction

Biogenic volatile organic compounds (BVOCs) are emitted from vegetation, including isoprenoids (isoprene and terpenes), aldehydes, alcohols, esters, ethers, acids, alkanes, and carbonyls [1]. The emission of plant-emitted VOCs greatly exceeds the emissions from anthropogenic sources on a global scale [2]. BVOCs emissions are estimated to be 1–1.5 Pg C per year on a global scale [3], accounting for about 10% of carbon recently fixed through photosynthesis processes from plants [4]. Isoprene and monoterpene emission are a major contribution to the total BVOCs flux, being about 57% and 14%, respectively [3]. BVOCs may play a protective role against abiotic stress and biotic stress in vegetation, such as heat, drought, wounding, ozone exposure, and CO_2_ exposure [5,6,7].

Climate change is a global issue of concern, with characteristics of climate warming and precipitation pattern change directly impacting plant growth and carbon cycles in terrestrial ecosystems. Drought stress is a typical seasonal occurrence in many locations, but the impact of drought on BVOCs emission is controversial. Depending on the studied plant species, BVOCs emissions were found to be reduced [8,9], enhanced [10,11], or unchanged [12] in response to drought stress. Pegoraro et al. [13] found that the BVOCs emissions of *Quercus virginiana* were generally found to be reduced under severe drought stress. However, the research of Wu et al. [10] on *Pinus sylvestris* and *Picea abies*, and that of Dani et al. [11] on *Eucalyptus robusta*, suggested that isoprenoid emission increased under drought. In addition to changes in tree species, studies on the mechanisms of drought stress on BVOCs emissions are also scarce. Staudt et al. [14], who studied *Quercus suber* L. saplings, found that severe drought decreased monoterpene emissions to about two thirds of that of the controls. Ormeno et al. [12] observed in the VOC emissions from *Q. coccifera* a decrease in sesquiterpene and an increase in monoterpene emissions during an increasing water deficit. Yuan et al. [15] investigated the effect of moderate drought on the poplar hybrid clone 546, finding that drought-stimulating isoprene emissions (+384%), which increased with increasing A_sat_ and chlorophyll content, were largely independent of g_s_ and were strictly controlled by C_i_. Pegoraro et al. [16] found that moderate drought stress may stimulate isoprene emissions via low C_i_. According to Brilli et al. [17], who found opposite effects to those caused by a severe drought, drought impairs the photosynthetic process, thus leading to a decrease in photosynthetic assimilation and therefore, to a decrease in the bio-synthetic mechanism of isoprene formation. Nogués et al. [8] reported that drought stress reduced BVOCs emissions and that the release of BVOCs may be associated with photosynthesis, which may indicate that BVOCs emissions depend on photosynthesis and “de novo” synthesis. The effect mechanism of drought stress on BVOCs emissions is still unclear.

*Pinus massoniana* is the main pioneer afforestation tree species in South China, accounting for 20% of the total afforestation area in China. With extensive distribution and wide range of uses, *P. massoniana* plays an important role in timber production and environmental protection [18]. Under the background of global change, drought stress is a major abiotic stress event, which has a large impact on plant growth and physiology. Additionally, BVOCs play an important role in the resistance to such stress. However, there is a paucity of studies on BVOCs emission response to drought stress, based on BVOCs types, components, and physiological mechanisms. Therefore, we conducted a study on the changes in the BVOCs emission rates of three-year-old *P. massoniana* saplings in response to drought stress. The objectives of our study were (1) to determine the type of BVOCs emitted from *P. massoniana* under drought stress, (2) to quantify the emission rates of BVOCs in response to drought stress, and (3) to elucidate the physiological mechanisms of BVOCs emissions in response to drought stress.

## 2. Results

### 2.1. The Appearance Frequency of BVOCs Components in Response to Drought Stress

**Figure 1:**
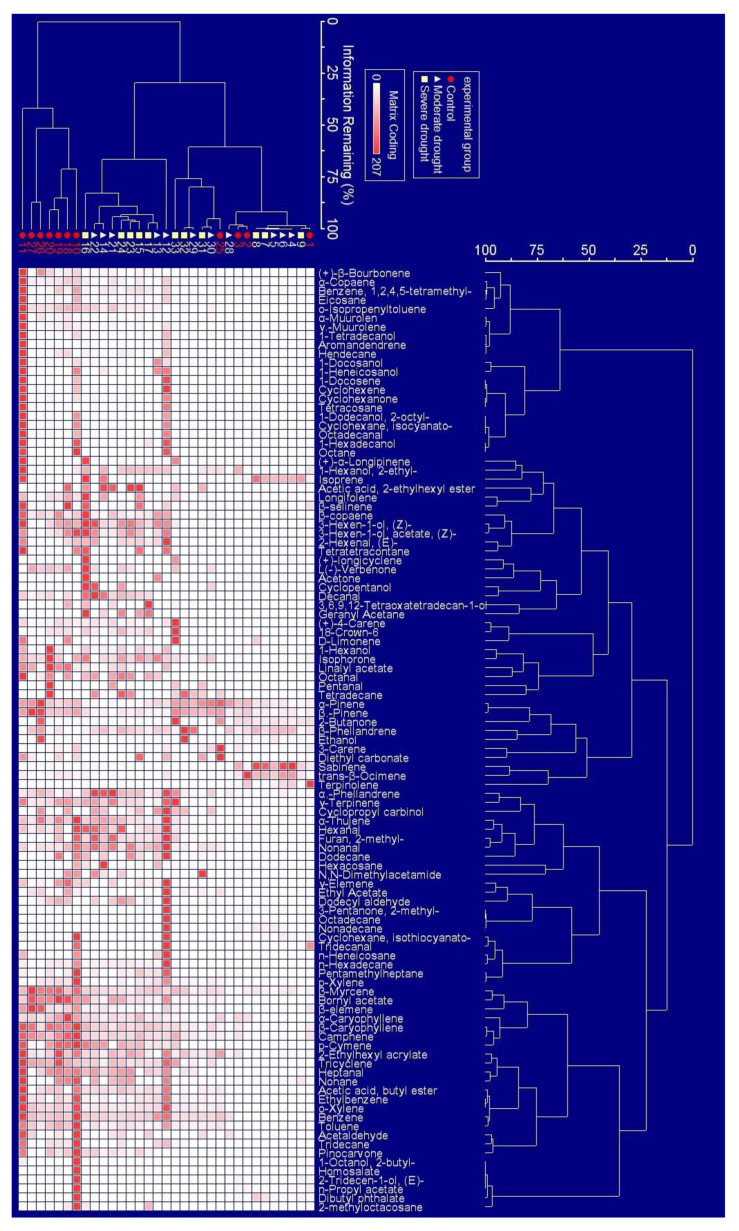
Two-way cluster analysis for the emissions rates of BVOCs components under drought stress. The emission rates were represented by the degree of color gradient. The emission rate of BVOC component increased as the color deepens.

The appearance frequency of the emission rates of BVOCs components under drought stress was visually evaluated using two-way cluster analysis. As shown in Figure 1, the appearance frequency of (+)-β-Bourbonene, α-Copaene, Benzene,1,2,4,5,-tetramethyl-, Eiicosane, ο-Isopropenyltoluene, α-Muurolen, γ-Muurolene, 1-Tetradecanol, Aromandendrene, and Hendecane were low (less than 27.28%), with almost no appearance frequency when *P. massoniana* saplings were under drought stress. The appearance frequency of the BVOCs components, such as β-Copaene, 3-Hexen-1-ol,(Z)-, 3-Hexen-1-ol, acetate,(Z)-, 2-Hexenal,€-, Cyclopentanol, Decanal, α-Phellandrene, γ-Terpinene, Cyclopropyl carbinol, α-Thujene, Hexanal, Furan,2-methyl-, and Nonanal, was high (more than 60%), both under drought treatment and in the control group. Similarly, their emission rates were high when plants were under drought stress. The appearance frequency of the BVOCs components, such as α-pinene, β-pinene, β-Myrcene, Bornyl acetate, β-Elemene, α- Caryophyllene, β-Caryophyllene, Camphene, ρ-Cymene, 2-Ethylhexyl acrylate, Tricyclene, Heptanal, and Nonane, was high (more than 80%), both under drought treatment and in the control group, but their emission rates declined under drought stress.

### 2.2. Drought Effect on BVOCs Emission Rate

**Figure 2:**
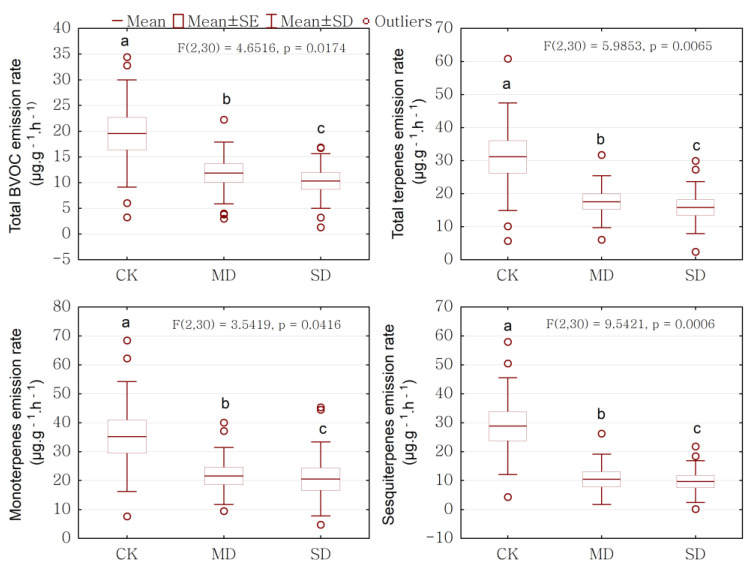
Drought effect on BVOCs emission rate. CK represents control, MD represents moderate drought, and SD represents severe drought in the horizontal axis. Significant differences (*p* < 0.05 using ANOVA) between treatments are indicated by different letters.

**Figure 3:**
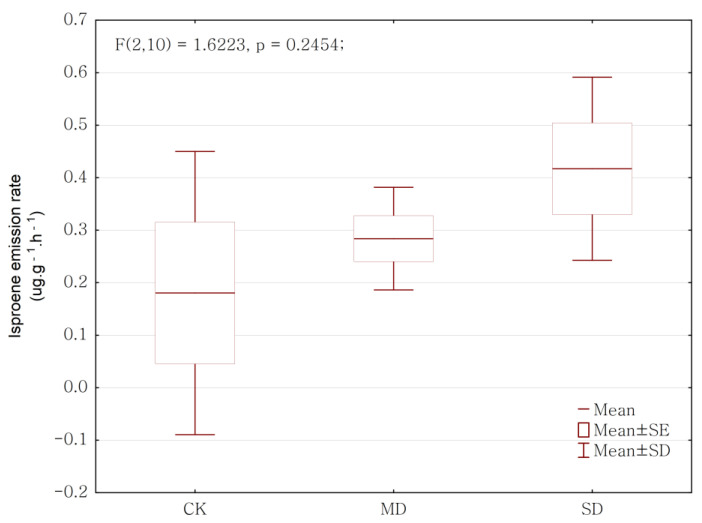
Drought effect on isoprene emission rate. CK represents control, MD represents moderate drought, and SD represents severe drought in the horizontal axis.

We observed a significant impact of the drought treatments on BVOCs emission rates (*p* < 0.05). Overall, *P. massoniana* sapling under drought stress exhibited significantly lower BVOCs emission rates. The emission rates of total BVOCs, total terpenes, monoterpenes, and sesquiterpenes were significantly affected by the drought stress (*p* < 0.05), but those of isoprene were not significantly affected by the drought stress (*p* > 0.05; ANOVA results shown in Table 1 and mean ± SD presented in Table 2). There were significant reductions in the emission rates of total BVOCs, total terpenes, monoterpenes, and sesquiterpenes from the control to the moderate drought and severe drought groups (Figure 2). There was a marginal increase in the emission rate of isoprene, but there was no significant difference (*p* = 0.2454, Figure 3). We observed that the emission rates of alcohol, aldehyde, and ketone did not obviously change under drought stress, while those of ester, alkane, oxygenated compounds, and aromatic were significantly reduced under drought stress (Table 2). The ratio of sesquiterpenes was significantly higher in the control than under moderate drought and severe drought. The ratio of monoterpenes was higher in the moderate drought group than in the severe drought and control groups, when there was not a significant difference in drought stress (Table 2). The compound components of the emitted monoterpenes changed under drought stress. The emission rates of α-Pinene, β-Pinene, β-Myrcene, and Camphene were significantly higher in the control group than the moderate drought and severe drought group. The emission rate of α-Phellandrene and β-Phellandrene did not change in a statistically significant way due to drought stress, but their emission rates were higher under drought stress than those in the control (Table 3). The compound components of the emitted sesquiterpenes were altered under drought stress. The emission rates of β-Caryophyllene and β-elemene were significantly higher in the control group, compared to the drought treatment (Table 3).

### 2.3. Overall Response of Physiology to Drought Stress

**Figure 4:**
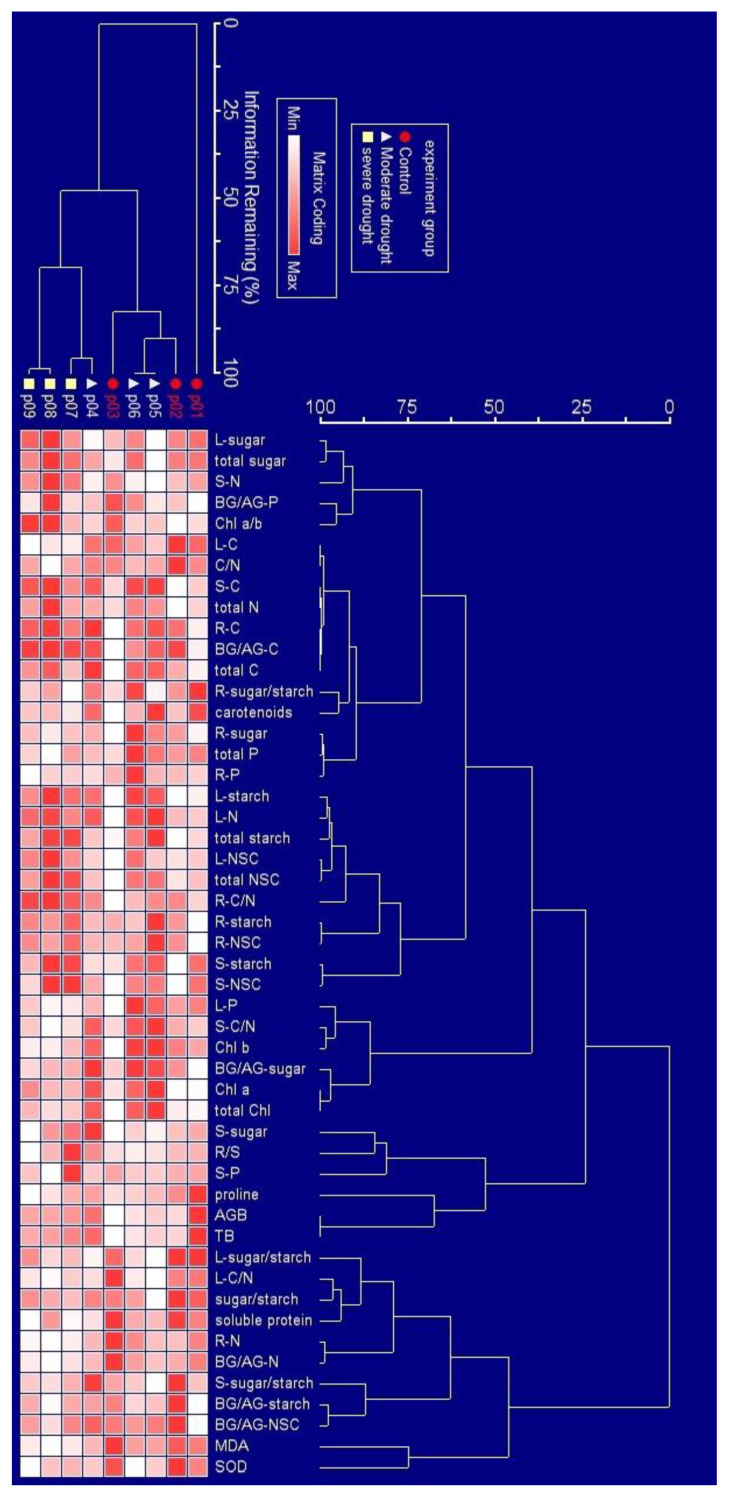
Two-way cluster analyses for the physiological parameter of *P. massoniana* saplings under drought stress. The relative values were represented by the degree of color gradient, increasing as the color deepens. Note: “L” in L-(sugar, starch, NSCs, sugar/starch, C, N, P, C/N) represents leaves; “S” in S-(sugar, starch, NSCs, sugar/starch, C, N, P) represents stems; “R” in R-(sugar, starch, NSCs, sugar/starch, C, N, P) represents roots; “total” in total-(sugar, starch, NSCs) means the whole plant; “BG/AG” means the ratio of belowground and aboveground; R/S = root shoot ratio = the ratio of belowground biomass and aboveground biomass, AGB = aboveground biomass, TB = total biomass of whole plant, Chl a/b = the ratio of Chlorophylls a and Chlorophylls b, Chl a = Chlorophylls a, Chl b = Chlorophylls b, total Chl = total Chlorophylls (Chlorophylls a + b).

The physiological parameter of *P. massoniana* saplings under drought stress are presented in Figure 4. The NSC and starch contents in different tissues (e.g., leaves, stems, and roots) and in the whole tree were increased as the extent of drought increased. The sugar content in the leaves, N content in the stems, and C content in the roots were highest under severe drought, being higher than that in the moderate drought and control groups. The chlorophylls content, including chlorophylls a, chlorophylls b, and total chlorophylls content, notably increased when the drought was moderate, but decreased when the drought became severe.

### 2.4. Physiological Response to Drought Stress

**Figure 5:**
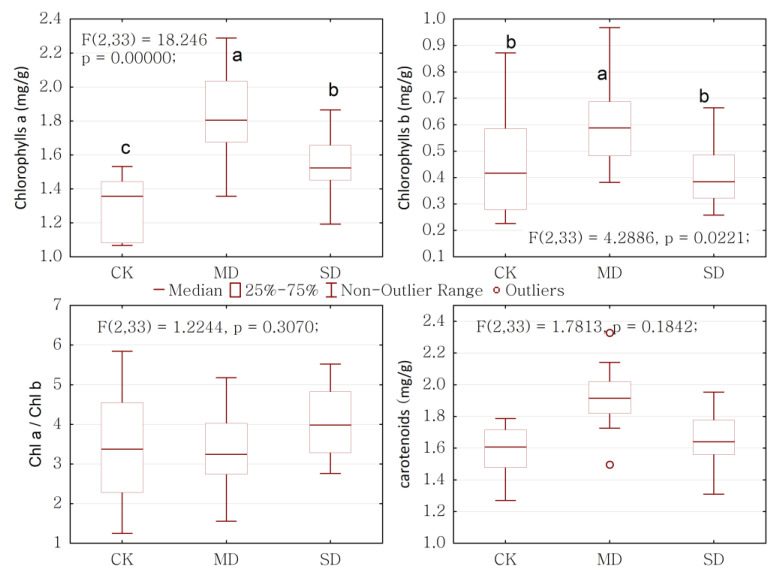
Chlorophylls and carotenoids of *P. massoniana* saplings under drought stress. Significant differences (*p* < 0.05 using ANOVA) between treatments are indicated by different letters. CK represents control, MD represents moderate drought, and SD represents severe drought in the horizontal axis.

The changes in chlorophylls and carotenoids under drought stress were investigated. As shown in Figure 5, chlorophylls a content was observed to demonstrate a significant difference under drought stress, notably increasing when the drought was moderate, but decreasing when the drought became severe. The change trend of chlorophylls b content was consistent with that of chlorophylls a, increasing significantly when measured under moderate drought stress, and declining under severe drought stress. There were no obvious upward or downward trends in chlorophylls a/b under drought stress. There was no significant difference in carotenoid content under drought stress, and it was highest under moderate drought stress (Figure 5).

**Figure 6:**
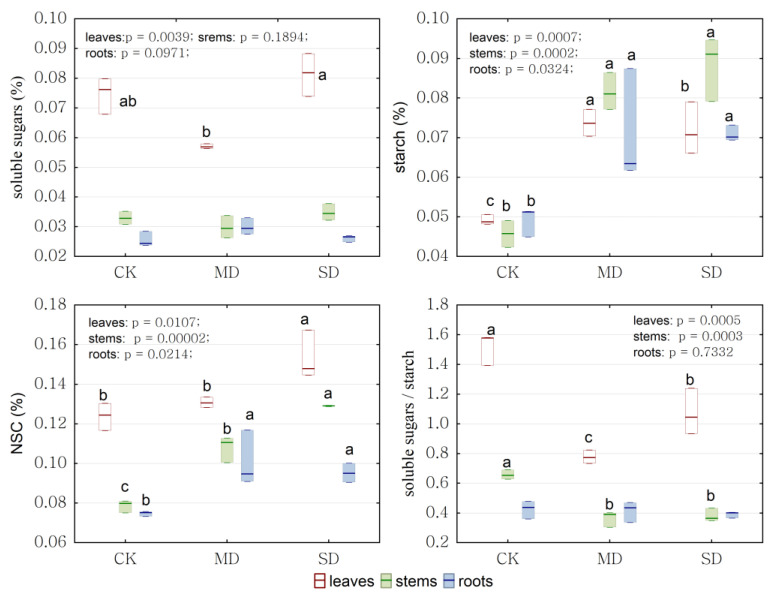
The soluble sugars, starch, non-structural carbohydrates (NSCs, soluble sugars + starch) content and the soluble sugars/starch in leaves, stems, and roots of *P. massoniana* saplings under drought stress. Significant differences (*p* < 0.05 using ANOVA) among treatments are indicated by different letters. CK represents control, MD represents moderate drought, and SD represents severe drought in the horizontal axis. White background boxes indicate leaves, green background boxes indicate stems, and blue background boxes indicate roots.

The impact of drought stress on soluble sugar, starch, and non-structural carbohydrates in different tissues was assessed. Drought stress had a significant effect on the soluble sugar content in leaves but had no significant effect on that in stems and roots. The soluble sugar content in leaves was higher than that in stems and roots, both in the drought stress and control groups. Drought stress had a significant effect on starch content in leaves, stems, and roots, consistently and significantly increasing under drought stress. The NSC content in all tissues obviously differed under drought stress. The NSC content in leaves and stems was highest in the severe drought group, being significantly higher than that in the moderate drought and control groups. The NSC content in roots under drought stress was higher than that in the control group. Both in the control and drought stress groups, the NSC content was mainly stored in leaves, which was higher than that in stems and roots. The soluble sugar/starch in leaves and stems significantly reduced under drought stress, but there was no obvious upward or downward trends in the soluble sugar/starch in roots under drought stress (Figure 6).

**Figure 7:**
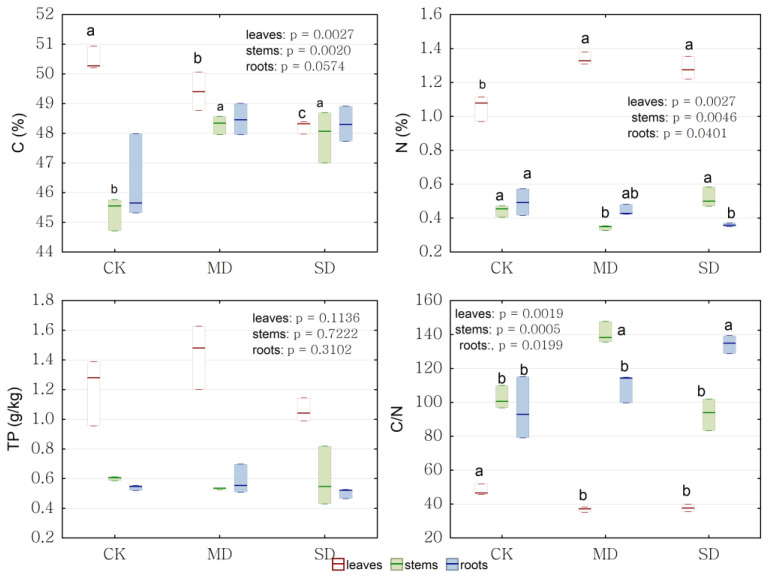
The C, N, P content, and the C/N in leaves, stems, and roots of *P. massoniana* saplings under drought stress. Significant differences (*p* < 0.05 using ANOVA) among treatments are indicated by different letters. CK represents control, MD represents moderate drought and SD represents severe drought in the horizontal axis. White background boxes indicate leaves, green background boxes indicate stems, and blue background boxes indicate roots.

The changes in carbon, nitrogen, and phosphorus content under drought stress in different tissues were quantified. There were significant differences in C content, N content, and the C/N in all tissues among the treatments, but there were no obvious changes in the P content in all tissues under drought treatment. The C content in leaves significantly decreased under drought stress. In contrast, the C content in stems significantly increased under drought stress; the C content in roots increased under drought stress, but there was no significant difference. The N content in leaves significantly increased under drought stress and the N content in stems and roots significantly decreased under moderate drought. Drought stress significantly decreased the C/N in leaves and increased the C/N in roots compared with the control. The C/N in stems significantly increased under moderate drought and significantly reduced under severe drought. The N content and P content in leaves were higher than those in stems and roots, both in the control and under drought stress, but the C/N in leaves was lower than that in stems and roots, both in the control and under drought stress (Figure 7).

**Figure 8:**
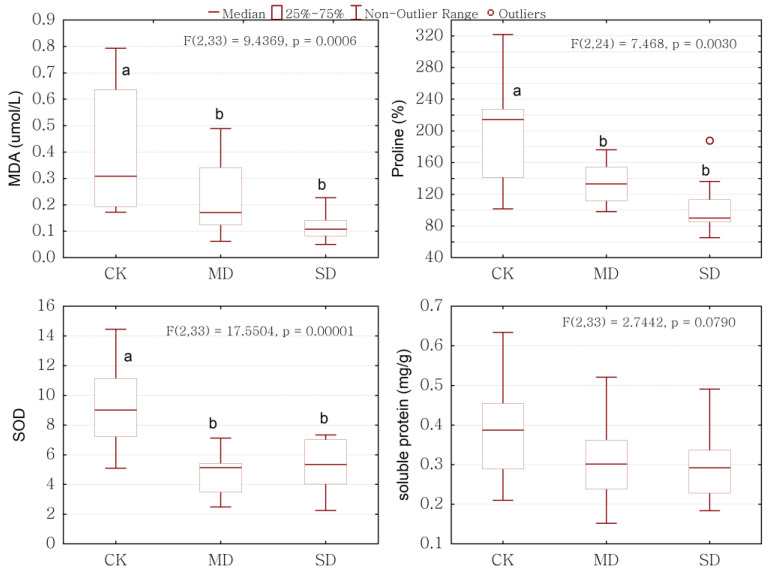
Changes in Malondialdehyde (MDA), Superoxide dismutase (SOD), proline, and soluble protein of *P. massoniana* saplings under drought stress. Significant differences (*p* < 0.05 using ANOVA) among treatments are indicated by different letters. CK represents control, MD represents moderate drought, and SD represents severe drought in the horizontal axis.

The oxidative damage, estimated by the peroxidation of leaf membrane lipids (MDA), was clearly decreased under drought stress, as it decreased substantially as the drought stress progressed. The activity of SOD and proline decreased significantly under moderate drought but decreased slightly under severe drought. The soluble protein content in leaf reduced under drought stress but did not vary significantly between the drought stress and control groups (Figure 8).

### 2.5. BVOCs Emission Associated with Physiological Traits

**Figure 9:**
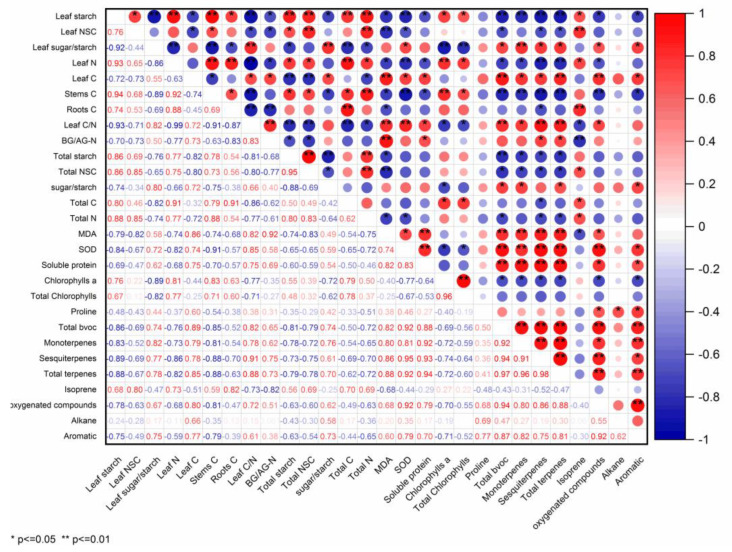
Correlations (r) between BVOC emission rate and physiological parameter.

BVOCs emission rates were closely associated with the physiological traits of *P. massoniana* saplings. The emission rates of total BVOCs, monoterpenes, sesquiterpenes, total terpenes, oxygenated compounds, and aromatic showed significant negative correlations with chlorophyll a, the starch content in leaves, and the C content in stems during the experiment period, while positive correlations were found between the isoprene emission rate and chlorophyll a, the starch content in leaves, and the C content in stems during the experiment period. A significantly positive correlation was detected between the isoprene emission rate and starch content in leaves. The emission rates of total BVOCs, monoterpenes, sesquiterpenes, and total terpenes were negatively correlated with leaf NSCs, total starch, total NSCs, leaf N, and total N, while the isoprene emission rate was positively correlated with the physiological traits. Statistically significant positive correlations were observed during the experiment period between the emission rates of total BVOCs, monoterpenes, sesquiterpenes, and total terpenes and soluble protein, MDA, SOD, the sugar/starch in leaves, the sugar/starch in the whole plant, leaf C, and the C/N in leaf. By contrast, a negative correlation emerged between the isoprene emission rate and the physiological traits. For the *P. massoniana* sapling, a significantly positive correlation existed between the isoprene emission rate and C content in roots and in the whole plant during the experiment period, but a significantly negative relationship was found between the sesquiterpenes emission rate and the C content in roots and in the whole plant during the experiment period (Figure 9).

**Figure 10:**
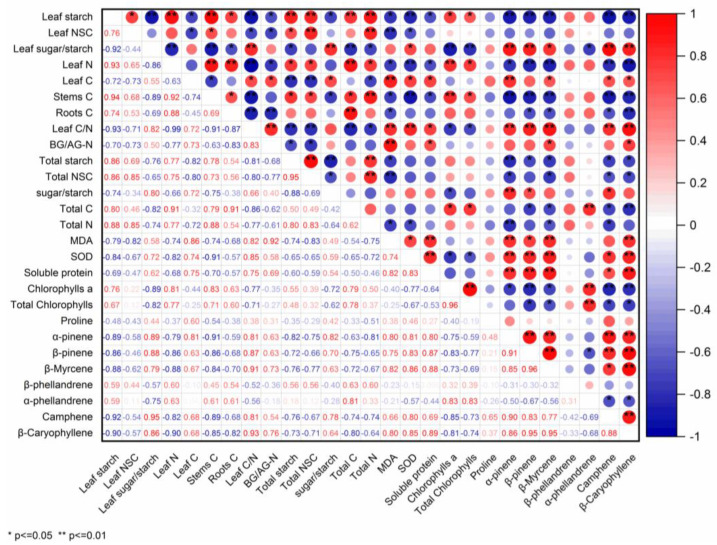
Correlations (r) between the emission rates of the most abundant released monoterpenes and sesquiterpenes and physiological parameter.

The emission rate of β-pinene, β-Myrcene, Camphene, and β-Caryophyllene were significantly negatively related to the chlorophylls a, total chlorophylls, and C content in the whole plant, while the α-phellandrene emission rate was significantly and positively related to the chlorophylls a, total chlorophylls, and C content in the whole plant. Statistically significant negative correlations among the emission rates of α-pinene, β-pinene, β-Myrcene, Camphene, and β-Caryophyllene and leaf starch, total starch, and NSC in the whole plant, as well as with the N content in leaves and the C content in stems, were observed during the experiment period. For the *P. massoniana* sapling, the emission rates of the most abundantly released monoterpenes and sesquiterpenes, including α-pinene, β-pinene, β-Myrcene, Camphene, and β-Caryophyllene, decreased with increasing leaf starch, total starch, and NSCs of the whole plant, as well as with increasing N content in leaves and C content in stems during the experiment period. Significant positive relationships between the emission rates of α-pinene, β-pinene, β-Myrcene, Camphene, and β-Caryophyllene and soluble protein, MDA, SOD, and the C/N in leaf were found during the experiment period, while a negative correlation emerged for the emission rates of α-phellandrene and β-phellandrene. The relationships between sugar/starch in leaves as well as in the whole plant and the emission rates of α-pinene, β-pinene, β-Myrcene, and Camphene were significantly positive, but they were negative for the emission rates of α-phellandrene and β-phellandrene (Figure 10).

## 3. Discussions

### 3.1. BVOCs Emission in Responses to Drought Stress

BVOCs improve the plant resistance pathway directly in their role as anti-stress substances and indirectly through signaling [19]. The *P. massoniana* trees emit BVOCs, allowing direct and indirect signal regulation when they suffer environmental stress [20]. In *P. massoniana* saplings, we identified α-pinene, β-pinene, α-phellandrene, β-phellandrene, and β-Myrcene as the principal monoterpenes, and β-elemene, longifolene, and caryophyllene as the most important sesquiterpenes (Table 3), which is consistent with previous research that reported the chemical components of needle essential oils of 46 pine species [21].

Environmental factors, such as water availability, play an important role in the emission of BVOCs from plants, and scientists have conducted numerous studies on these factors. Our results from the *P. massoniana* saplings showed that drought stress decreased BVOCs emission rates (Figure 2). Under our experimental conditions, BVOCs emission rates decreased to 40.28% and 47.6% under moderate drought stress and severe drought stress, respectively, compared with the control. At the same time, monoterpenes emission rates decreased to 39.37% and 37.33%, and sesquiterpenes emission rates accounted for a 55.40% and 60.34% reduction when stress was moderate and severe, respectively, compared with the control. Baker et al. [22] studied the tropical *Hevea brasiliensis* and Plaza et al. [23] studied *Quercus ilex* in Spain, with both finding a more than 50% reduction in monoterpene emissions under drought stress. Our results show that drought stress reduced BVOCs emission rates, including those of total BVOCs, monoterpenes, and sesquiterpenes (Figure 2 and Table 1), which was consistent with the results of previous studies that showed that under severe drought stress, BVOCs emissions were generally reduced [13]. Lavoir et al. [24] reported that monoterpene emissions from *Q. ilex* decreased under drought, Nogués et al. [8] showed that drought stress decreased the percentage of α-pinene and camphene released from *Rosmarinus officinalis*, and Staudt et al. [14] found that severe drought (20–30% of field water capacity) reduced the emission of monoterpenes from *Quercus suber* saplings to about 2/3 of the control group. However, the effect of drought stress on BVOCs emissions was controversial. Ormeno et al. [12] investigated the monoterpenes and sesquiterpenes emissions of four Mediterranean species during 11 days of water withholding and found that the monoterpene emissions of water stressed plants (t_1_–t_11_) were either similar to those of control seedlings (*R. officinalis* and *Q. coccifera*) or higher (*P. halepensis* and *C. albidus*). Blanch et al. [25] found that drought stress (a reduction to 1/3 of full watering) significantly increased the total terpene concentrations in both species (54% in *Pinus halepensis* and 119% in *Quercus ilex*).

Isoprene, as the most representative BVOCs emitted by plants, represents as much as 80% of the carbon emitted from plants [26,27]. Our results from *P. massoniana* saplings show that drought stress had no significant effect on the isoprene emission rate, and that there was a slight upward trend (Figure 3). These findings corroborate results from Wu et al. [10], who stated that drought increased isoprene emission in *Pinus sylvestris* and *Picea abies*, and Dani et al. [11] who stated that drought increased isoprene emission in *Eucalyptus robusta*. Yuan et al. [15] obtained similar results that drought stimulated isoprene emission (+38.4%). A change in any of these components may be important for the defense of *P. massoniana* saplings against drought stress. It seems that the effects of drought stress on BVOCs emissions vary based on the BVOCs components, tree species, and other factors (i.e., different plant tissues). These different responses may be linked either to different effects of drought on the particular synthesis of BVOCs components, or to possible different protective roles for different BVOCs components in the face of drought.

### 3.2. Physiological Mechanism of BVOCs Emission under Drought Stress

Some research has shown that *Pinus* genus plants use diverse, multifunctional biosynthetic pathways to produce BVOCs to collectively improve their defense when they suffer from biotic or abiotic stress [28,29]. BVOCs play an important role in the defense system of conifers against stress [21]. The changes in BVOCs components emissions are the results of the joint influence of the environment and plants, and physiological activities of plants internally play an important role in BVOCs component emissions. BVOCs, especially monoterpenes and isoprene, are produced in chloroplasts [30]. Previous studies reported that the isoprene emitted from plants is made in chloroplasts from dimethylallyl pyrophosphate (DMAPP) through the 2-Cmethyl D-erythritol 4-phosphate (MEP) pathway [31,32]. The MEP pathway produces many key compounds involved in the synthesis and maintenance of the photosynthetic apparatus (e.g., carotenoids, plastoquinone and chlorophylls), as well as antioxidant molecules [33]. The MEP pathway provides substrate for the synthesis of numerous terpenoids, in addition to isoprene, and has been implicated in the regulation of their synthesis [34]. Isoprene synthase appears to be localized to the chloroplasts; it has been found that, isoprene promoter activity (and isoprene synthesis) and photosynthetic capacity were greatest where chlorophyll content was also highest [35,36]. Our study showed that drought stress significantly increased the contents of chlorophyll a and chlorophyll b (Figure 5) and improved the photosynthetic capacity of *P. massoniana* saplings to adapt to the sharp decline in metabolites caused by drought stress. The contents of chlorophyll had a positive correlation with isoprene and phellandrene, providing a substrate for the synthesis of isoprene and phellandrene (Figure 9 and Figure 10). The research by Yuan et al. [15] also confirmed that isoprene emissions at the leaf level increased with increasing chlorophyll content. Therefore, the photosynthesis process and photosynthetic apparatus are important factors affecting BVOCs synthesis and emissions.

Our experiment indicated that drought stress decreased the emission of BVOCs released from *P. massoniana* saplings (Figure 2). This emission decrease is essentially explained by the reduced availability of primary carbon substrates necessary for BVOCs biosynthesis [37,38]. Lavoir et al. [24] reported that monoterpene emissions from *Q. ilex* decreased under drought and that this phenomenon was associated with the reduced availability of primary carbon substrates necessary for monoterpene biosynthesis. Large proportions of BVOCs releasing from Pinaceae are likely emitted from resins, but there is also evidence that BVOCs could originate directly from de novo synthesis [39]. Under drought stress, because of a low carbon supply, stored compounds, such as NSCs, and newly assimilated carbon, can provide substrates for BVOC production [6]. A previous study showed that carbon fixed through photosynthesis was used to synthesize BVOCs [40]. Drought stress also causes leaf stomata closure and photosynthetic limitations, which were expected to negatively affect the carbon supply into the MEP pathway and reduce BVOCs biosynthesis [30,41]. Huang et al. [42] showed that spruce saplings exposed to a low carbon supply invest carbon into monoterpenes at the cost of storage and growth. Funk et al. [43] indicated that in addition to the direct use of photosynthetically fixed carbon, isoprene formation is controlled by the whole-plant carbon allocation pattern. In our experiment, a positive relationship was observed between the emission rates of the total BVOCs, terpenes, monoterpenes, sesquiterpenes, and oxygenated compounds and the sugar/starch of *P. massoniana* saplings under drought stress, while a negative relationship was observed between the isoprene emission rate and the sugar/starch of *P. massoniana* saplings under drought stress. Conversely, a negative relationship of the leaf starch, leaf NSCs, total starch, and total NSC of the whole plant with the emission rates of the total BVOCs, terpenes, monoterpenes, sesquiterpenes, and oxygenated compounds was observed, while a positive relationship was shown with the isoprene emission rate (Fiigure 9). Previous studies confirmed that under drought stress, isoprene synthesis in plants continues via alternative sources of carbon earlier fixed, and carbon stores have been possibly identified to be related to starch breakdown through carbon labeling experiments [30,41]. Schnitzler et al. [44] suggested that in addition to xylem transported carbon, leaf internal carbon pools, e.g., starch, are used for isoprene formation. Therefore, carbon substrates for BVOCs biosynthesis are not only from the new carbon obtained through photosynthesis, but also from the carbon stored by plants, which is related to the carbon distribution pattern of the whole-plant.

Drought can result in oxidative stress, which needs to be compensated by an antioxidant system or other defenses in order to avoid injury. The SOD activity is closely related to the resistance of plants themselves. It is a major protective enzyme in plant cells that resists the damage of reactive oxygen species (ROS) and plays an important role in clearing and preventing the production of ROS [45]. Our results showed that the SOD activity significantly decreased under drought stress (Figure 8), indicating that drought stress broke the dynamic balance of production and the elimination of free radicals in plant cells, and resulted in the excessive accumulation of active oxygen, which exceeded the scavenging capacity of the protection system; thus, there was a downward trend. The soluble protein content is an important osmotic regulator and nutrient in plant tissues [46]. Under drought conditions, plant cells will accumulate osmoregulation substances, such as soluble proteins, to reduce the cell osmotic potential, increase the cell water capacity, and maintain the normal physiological activities of plants [47]. Our study showed that the soluble protein content decreased, but not significantly, under drought stress; our results were different from that of other research, which showed that the soluble protein content increased under drought stress [48]. This could be attributed to the limited capacity of plants to adapt to drought stress by synthesizing osmoregulation substances, such as soluble proteins. Under drought stress, the osmotic regulation system gradually becomes disordered, and the content of soluble proteins in plant tissues also decreases. BVOCs play a protective role against drought stresses. BVOCs emissions are one of the defenses available to plants to deal with oxidative stress. We also found that the BVOCs emission rate was significantly positively related with SOD activity and soluble protein content (Figure 9 and Figure 10). In our study, *P. massoniana* saplings emitted isoprene under drought stress to resist promoting oxidative stress. It has been proposed that isoprene may physically stabilize membranes and/or behave as an antioxidant, presumably preventing lipid peroxidation and minimizing the oxidative damage to membranes [49,50,51].

## 4. Materials and Methods

### 4.1. Plant Material and Experimental Design

Forty-five three-year-old *Pinus massoniana* Lamb plants (H = 165.24 ± 30.14 cm, DBH = 14.92 ± 2.53) were planted in 19 L pots filled with soil collected from a field, with the initial properties as follows: soil organic carbon, 19.55 ± 1.19 g·kg^−1^; soil total nitrogen, 1.42 ± 0.13 g·kg^−1^; soil total phosphorus, 0.27 ± 0.01 g·kg^−1^; soil available P, 0.63 ± 0.23 mg·kg^−1^; soil available potassium, 47.15 ± 3.44 mg·kg^−1^; and pH, 4.61 ± 0.3. These plants were planted in a greenhouse from July to November 2020, which was located at the Qianjiangyuan Forest Ecosystem Research Station (Fuyang district, Hangzhou, China, 119.56° E, 30.06° N). All the collected plant materials were divided into 3 treatments, with 15 plants in each treatment, including the control (CK, maintained 80–85% of the maximum field moisture capacity), moderate drought (MD, 50–55% of the maximum field moisture capacity), and severe drought (SD, 30–35% of the maximum field moisture capacity) group. All plants were grown under the same growing conditions except for the water conditions. The BVOCs samples were collected 4 times across the experiment period, with sampling occurring at 30 days, 45 days, 60 days, and 75 days after the drought treatment was applied. Tree samples were taken each time.

### 4.2. Physiological and Biochemical Characteristics Analysis

After collecting BVOCs samples, the whole plant, including the roots, stems, and leaves, was harvested and stored separately, and brought back to the laboratory for analysis. Fresh leaves were used for the determination of chorophyll and enzyme activities. In addition, the contents of nonstructural carbohydrates C, N, and P were determined after the plant samples dried at 70 °C to a constant weight. Malondialdehyde (MDA) was used as a marker to quantify the leaf lipid peroxidation. The concentration of MDA was measured spectrophotometrically on leaves using the thiobarbituric acid (TBA) reaction [52]. Superoxide dismutase (SOD) activity was measured with ultraviolet spectrophotometry at 560 nm, based on the inhibition of nitroblue tetrazolium (NBT) reduction by SOD [53]. One unit of SOD was defined as the enzymatic amount needed to reduce the NBT reduction state by 50%. Chlorophyll and carotenoid contents were measured with spectrophotometry at 645, 470, and 663 nm, and were calculated according to the method of Lichtenthaler [54].

Nonstructural carbohydrates (NSCs) facilitate the adaptation of trees to drought stress. NSC changes in individual plant species and individual tissues under drought show different trends. In this study, the concentrations of soluble sugar, starch, and NSCs in different tissues were measured to evaluate their responses to drought intensity. All plant tissues samples were ground with a ball mill (JXFSTPRP-24, Shanghai, China) after over drying for 72 h at 70 °C. The soluble sugar and starch were measured using anthrone colorimetry and the content of tissue NSCs was calculated as the sum of soluble sugar and starch concentrations for each tissue [55].

Carbon (C), nitrogen (N), and phosphorus (P) are the most important elements in the component of organisms, and the content and distribution of C, N, and P in a plant reflect its response and adaptation to environmental changes. Elemental analysis (Vario EL III, Elementar Analyzer Systeme GmbH, Hanau, Germany) was used to measure the percentage of carbon in different plant tissues. The indophenol blue colorimetric method on a fully automatic nitrogen analyzer was employed to determine the nitrogen content and molybdenum antimony anti colorimetry was used to determine the content of total phosphorus in different parts of the pine plant. The C/N ratio was calculated as the ratio of carbon content to nitrogen content.

### 4.3. Biogenic Volatile Organic Compound (BVOCs) Sampling and Analysis

BVOCs were sampled from leaves and stems during the experiment period, and the values of BVOCs emissions were determined four times to define the average value. The whole saplings were used as samples. They were trapped with a Teflon bag and connected with the atmospheric sampler (QC-1B) via a Teflon tube, forming a closed loop of blanches and the sampling device. BVOCs were collected via a steel tube filled with 200 mg of Tenax (mesh 60/80) for 15 min at a flow rate of 400 mL min^−1^. Then, the sample tubes were sealed with brass caps immediately after the sampling and were stored at 4 °C until the analysis. Empty chamber blanks were also collected under the same chamber conditions in order to acknowledge and remove any background contamination. After BVOCs sampling, the whole plant was harvested to analyze its physiological and biochemical characteristics.

The analyses of BVOCs were performed with a gas chromatography-mass spectrometry device (GC/MS, GC7890-MSD5975C, Agilent, Santa Clara, CA, USA), which was equipped with an automatic sampled injection thermal desorption instrument (TD-100). Volatiles were analyzed using the parameters as follows: The BVOCs were first thermodesorbed at 250 °C for 5 min and then separated using an HP-5MS capillary column (30 m × 0.25 mm, film thickness 0.25 μm, Agilent, USA). The carrier gas was helium. The column gas flow rate was 1 mL min^−1^. The temperature of the column was first maintained at 35 °C (3 min) and then was increased with a 4 °C min^−1^ ramp to 160 °C and maintained at 160 °C for 2 min. Finally, it was increased further to 280 °C at a rate of 25 °C and was maintained at 280 °C for 4 min. BVOCs identification was based on mass spectrometry, using the NIST library provided with the GC/MS Turbomass software. The peak retention time and the peak fragmentation of an authentic standard were also used to positively identify BVOCs. The matching degree was higher than 90%. The compounds analyzed were summarized into six volatile organic compound groups consisting of isoprene, monoterpenes (MTs), sesquiterpenes (STs), oxygenated compounds (OCs), Alkane, and Aromatic. Since the environmental conditions varied during samplings, all experimentally determined emission rates were normalized to standard conditions (30 °C temperature and 1000 umol/m^2^ s PAR). The BVOCs emission rates were standardized according to the temperature- and light-dependent model established by Guenther et al. [56], which is widely used to assess BVOCs emission fluxes from plant leaves [57,58].

### 4.4. Statistical Analysis

To explore the physiological mechanisms of the BVOCs emission of the *P. massoniana* plant in response to drought stress, BVOCs emission rates and physiological parameters due to water stress were detected. Analyses of variance (ANOVA) were performed to assess the effect of drought stress on the emission rates of BVOCs components, using the Duncan post hoc test to investigate the significance of different groups, where *p* < 0.05 was considered significant. Pearson correlation was used to assess the relationships between physiological characteristic and BVOCs emission rates. A two-way cluster was performed using the relative values of all physiological traits and BVOCs component emission rates to comprehensively evaluate the differences in the plant physiological responses and BVOCs emission rates under water stress, respectively. In order to understand the emission of BVOCs response to drought stress, we calculated the percentage of reduction in BVOCs emission of Pinus massoniana under drought stress, which was calculated by (E0 − Ei)/E0 × 100%, where E0 represents the emission rate of BVOCs in the control group and Ei represents the emission rate under moderate and severe drought groups. All statistical analyses were conducted using Statistica 12.0, except for the two-way cluster, which was conducted using PC-ORD.

## 5. Conclusions

This work was performed to explore the effect of drought stress on the emission of BVOCs components and the physiological mechanism of BVOCs emission of *P. massoniana* saplings. Our results revealed that drought significantly decreased the emission rates of total BVOCs, total terpenes, monoterpenes, sesquiterpenes, oxygenated compounds, and aromatic, but slightly increased the isoprene emission rate. The emission rates of the most abundantly released monoterpenes, such as α-Pinene, β-Pinene, β-Myrcene, and Camphene, were significant declined according to the degree of drought. A significant negative relationship between the emission rates of total BVOCs, monoterpenes, and sesquiterpenes and chlorophylls, starch, NSCs, leaf N, total N, and stems C was observed; however, the emission rate of isoprene was contrary. The emission rates of α-Pinene, β-Pinene, β-Myrcene, Camphene, and β-Caryophyllene were significantly negatively correlated with chlorophylls, starch, NSCs, leaf N, total N, and stems C, and the emission rate of α-phellandrene and β-phellandrene were positively related to chlorophylls, starch, NSCs, leaf N, total N, and stem C. These results indicate a different control mechanism for the emission of the different components of BVOCs. Under drought stress, the emission trade-off between isoprene and other BVOCs components may be driven by the content of chlorophylls, starch, and NSCs. Our research enriches our knowledge of the emission of BVOCs components of different tree species under drought stress and reveals the possible mechanism of BVOCs emission through the physiological aspects of the content of chlorophylls, starch, and NSCs.

## Figures and Tables

**Table 1:** ANOVA analysis for the drought effects on the emissions rate of BVOCs.

Variables	SS	df	MS	F	*p*
Total BVOC	538.08	2	269.04	4.65	0.017
Total terpenes	1552.38	2	776.19	5.99	0.006
Monoterpenes	1469.33	2	734.67	3.54	0.042
Sesquiterpenes	2590.79	2	1295.39	9.54	0.001
Isoprene	0.112834	2	0.056417	1.622	0.245

**Table 2:** Changes in the emissions rate of total biogenic volatile organic compounds, the ratios of MT, ST, and OCs to the total emission of BVOCs during drought stress. Data represent the mean ± SD. Significant differences (*p* < 0.05 using ANOVA) are indicated by different letters. The oxygenated compounds were the sum of alcohol, aldehyde, ketone, and ester.

Variable	Control	Moderate Drought	Severe Drought
Total BVOC	19.56 ± 10.44 ^a^	11.88 ± 6.02 ^b^	10.32 ± 5.33 ^c^
Total terpenes	31.15 ± 16.29 ^a^	17.57 ± 7.87 ^b^	15.79 ± 7.87 ^c^
Monoterpenes (MT)	35.22 ± 19.01 ^a^	21.61 ± 9.85 ^b^	20.57 ± 12.82 ^c^
Sesquiterpenes (ST)	28.83 ± 16.73 ^a^	10.44 ± 8.69 ^b^	9.66 ± 7.19 ^c^
Isoprene	0.18 ± 0.27	0.28 ± 0.09	0.42 ± 0.17
Alcohol	17.63 ± 24.21	10.17 ± 12.63	7.85 ± 10.74
Aldehyde	9.81 ± 13.33	16.74 ± 25.67	11.42 ± 18.90
Ketone	9.05 ± 11.69	4.77 ± 6.61	8.59 ± 9.84
Ester	20.09 ± 25.26 ^a^	9.23 ± 12.67 ^b^	7.93 ± 11.14 ^c^
Alkane	12.20 ± 18.87 ^a^	10.14 ± 17.74 ^ab^	4.82 ± 9.24 ^b^
oxygenated compounds (OCs)	14.62 ± 20.68 ^a^	10.31 ± 16.44 ^b^	8.56 ± 13.63 ^c^
Aromatic	13.51 ± 20.74 ^a^	6.39 ± 10.11 ^b^	5.74 ± 10.82 ^c^
MT: total BVOCs (%)	48.13 ± 21.41	60.42 ± 31.59	55.12 ± 30.59
ST: total BVOCs (%)	20.90 ± 7.46 ^a^	9.24 ± 7.49 ^b^	9.95 ± 8.23 ^b^
OCs: total BVOCs (%)	21.99 ± 9.57	21.59 ± 17.66	26.88 ± 18.57

**Table 3:** Components of BVOCs emissions. The percentage of the six most abundant released monoterpenes and sesquiterpenes are listed. Data represent the mean ± SD. Significant differences (*p* < 0.05 using ANOVA) are indicated by different letters.

Variable	Compounds	Control	Moderate Drought	Severe Drought
Monoterpenes	α-Pinene	94.85 ± 53.01 ^a^	52.08 ± 42.19 ^b^	49.77 ± 40.41 ^c^
	β-Pinene	92.04 ± 56.83 ^a^	29.07 ± 29.09 ^c^	43.14 ± 37.39 ^b^
	β-Myrcene	68.32 ± 51.24 ^a^	23.27 ± 12.88 ^c^	27.29 ± 15.82 ^b^
	β-Phellandrene	27.73 ± 21.28	43.70 ± 22.92	36.39 ± 40.32
	α-Phellandrene	9.89 ± 10.42	33.32 ± 35.19	15.87 ± 16.02
	Camphene	54.65 ± 40.08 ^a^	14.36 ± 10.92 ^c^	19.75 ± 16.51 ^b^
Sesquiterpenes	α-caryophyllene	40.07 ± 33.60	14.87 ± 8.20	19.29 ± 12.21
	β-Caryophyllene	80.01 ± 62.01 ^a^	22.38 ± 18.24 ^c^	27.99 ± 22.23 ^b^
	β-Bourbonene	44.72 ± 42.39	6.72 ± 2.98	3.63 ± 2.41
	β-elemene	54.16 ± 39.91 ^a^	12.56 ± 10.31 ^b^	5.63 ± 2.21 ^c^
	Longifolene	6.03 ± 5.39	4.05 ± 6.04	7.13 ± 11.08

## Data Availability

Not applicable.
